# miR-20b and miR-125a promote tumorigenesis in radioresistant esophageal carcinoma cells

**DOI:** 10.18632/aging.202690

**Published:** 2021-03-10

**Authors:** Didi Chen, Huafang Su, Yunhao Li, Xinyi Wu, Yifei Li, Chaoyi Wei, Deli Shi, Ya Gao, Qingyu Zhou, Qiongqiong Wang, Xiance Jin, Congying Xie

**Affiliations:** 1 Department of Radiation and Medical Oncology, The First Affiliated Hospital of Wenzhou Medical University, Wenzhou, Zhejiang, China

**Keywords:** esophageal cancer, radiotherapy resistance, miR-20b-5p, miR-125a-5p, tumorigenesis

## Abstract

Radiation therapy is an effective method in the management of esophageal cancer. MicroRNAs (miRNAs) have been reported to play an important role in tumorigenesis. However, the roles of specific miRNAs in radioresistant esophageal cancer remain to be investigated. In present study, the relative expression level of miR-20b-5p and miR-125a-5p were evaluated by quantitative Real-time polymerase chain reaction. Cell counting Kit-8 assay, wound-healing assay, transwell assay were used to assess cell proliferation, cell migration and cell invasion. TUNEL and Annexin V-FITC assays were applied to evaluate cell apoptosis. Dual-luciferase reporter gene assay was conducted to identify direct targets of miRNAs. The protein expression level was assessed by Western blot. The results indicated that miR-20b-5p was increased in radioresistant KYSE-150R cells compared with KYSE-150 cells, whereas miR-125a-5p was downregulated. MiR-20b-5p upregulation promoted cell proliferation, migration, invasion, and the EMT process, and decreased apoptosis by negatively regulating PTEN. MiR-125a-5p inhibited cell proliferation, migration, invasion, the EMT process and it induced apoptosis by negatively regulating IL6R. These data indicate that miR-20b-5p and miR-125a-5p promote tumorigenesis in radioresistant KYSE-150R cells and have the potential to be used as novel therapeutic targets for the treatment of esophageal cancer.

## INTRODUCTION

Esophageal cancer (EC) is characterized by difficulties in early detection, a considerable decline in quality of life and poor prognosis. Radiotherapy is an important part of multidisciplinary treatment of EC. Chemoradiotherapy is recommended as a reasonable standard treatment option for unresectable esophageal squamous cell cancer patients [1]. Although today's three-dimensional conformal radiotherapy, intensity-modulated conformal radiotherapy increase the accuracy of radiotherapy, which improves the curative effect of EC patients. Acquired or innate radioresistance usually occurs, which is one of the major reasons for treatment failure and relapse [2]. The mechanisms of resistance to radiotherapy have not been fully understood. Previous studies indicated it may be caused by microenvironmental hypoxia, abnormal intrinsic DNA damage response activity, mutations of oncogenes or tumor suppressors or the altering of signaling pathways [3].

MicroRNAs (miRNA) are small non coding RNAs, which can not encode any protein. However, they can regulate gene expression by degrading messenger RNAs (mRNA) [4]. Accumulating evidence has indicated that miRNAs play a pivotal role in cancer formation and progression [5]. MiRNAs were also relevant to radioresistance in several cancers, such as nasopharyngeal carcinoma, laryngeal carcinoma and lung cancer [6–8]. For example, miR-20b is upregulated in several cancers, serves as an oncogene and is correlated with poor prognosis [9, 10]. MiR-125a is reduced in various cancer cells, which prevents cancer cell proliferation and induces apoptosis [11–13]. However, whether miR-20b and miR-125a affect tumorigenesis in radioresistant EC cells remains unknown.

In our previous reports, we had identified 35 miRNAs that were differentially expressed in radiation resistant EC cells, including miR-20b and miR-125a [14]. Here, we used KYSE-150R cells, which are verified radioresistance cell lines of EC, as a cellular model to assess the regulartory effect of two miRNAs on tumorigenesis of EC.

## RESULTS

### MiR-20b-5p was upregulated, and miR-125a-5p was downregulate in radioresistant EC cells KYSE-150R

**Figure 1:**
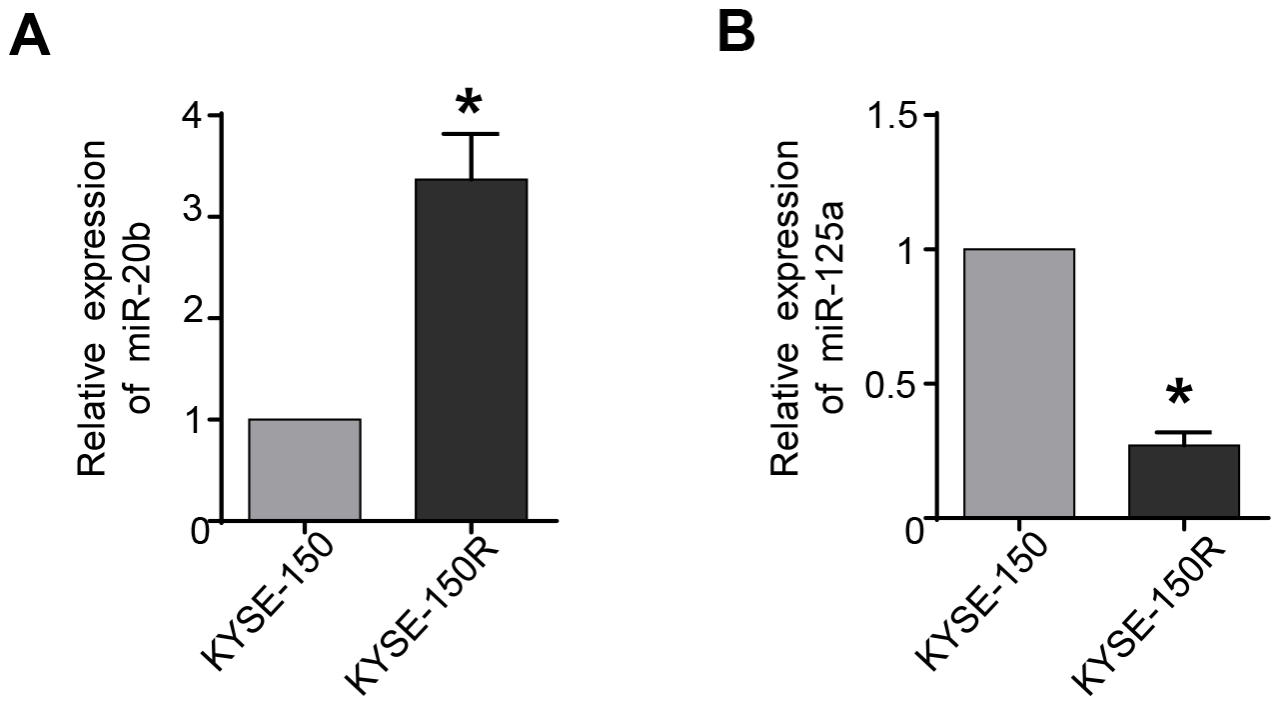
**miR-20b is upregulated and miR-125a is downregulated in radioresistant esophageal cancer cells KYSE-150R.** (**A**) The relative levels of miR-20b in parental (KYSE-150) cells and radioresistant (KYSE-150R) cells. (**B**) The relative levels of miR-125a in KYSE-150 cells and KYSE-150R cells. *P<0.05 by Student’s t-test.

The results of qRT-PCR analyses showed that expression levels of miR-20b-5p were significantly up-regulated in radioresistant EC KYSE-150R cells compared to those in the control EC KYSE-150 cells (Figure 1A). While the expression of miR-125a-5p was declined in KYSE-150R cells (Figure 1B). To dissect the biological function of miRNA, we planned to alter the expression of miR-20b-5p and miR-125a-5p in KYSE-150R cells and then investigate the cellular process after the change.

### MiR-20b-5p increases cell proliferation, migration, invasion, and EMT process, and attenuates cell apoptosis

**Figure 2:**
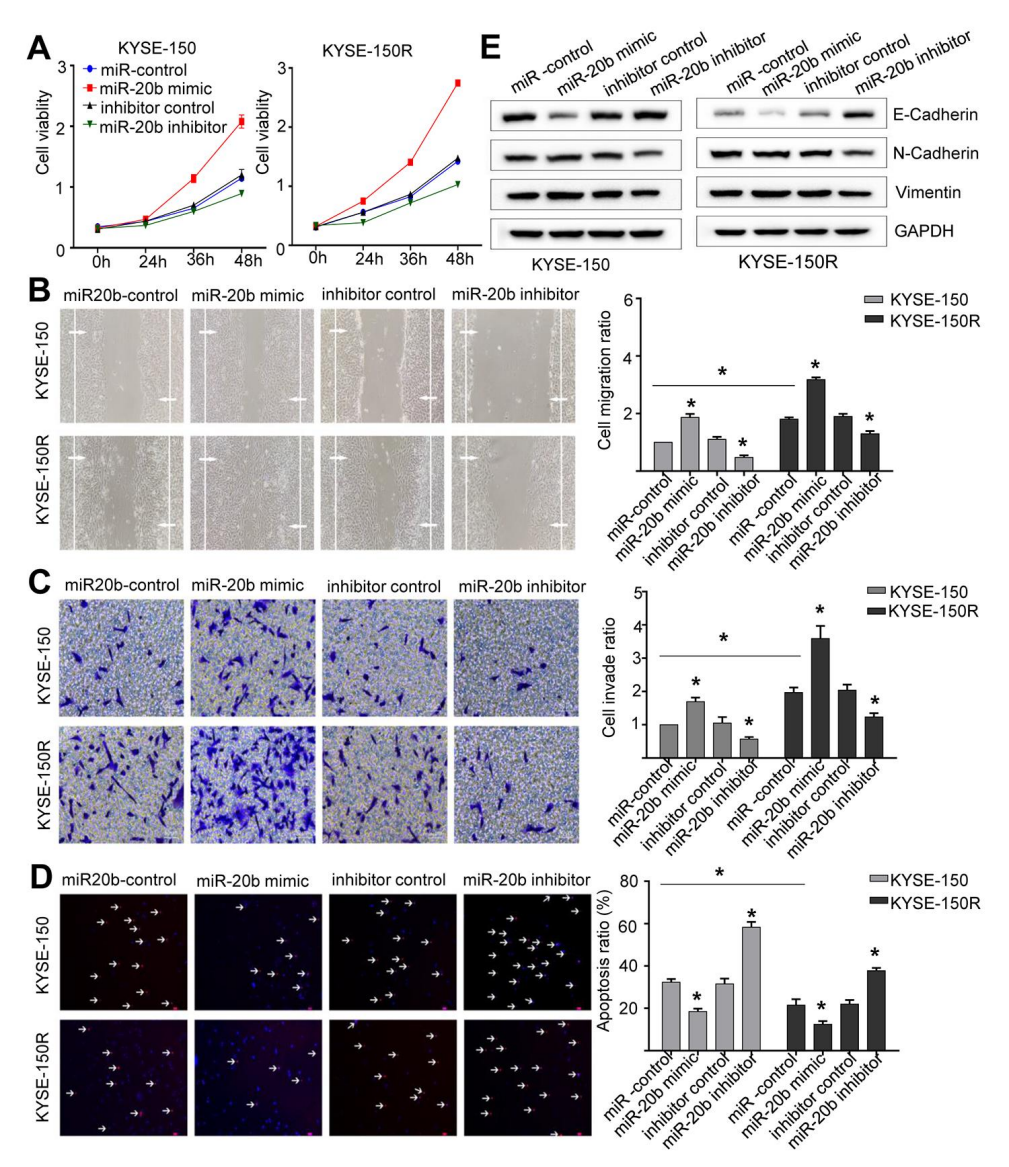
**miR-20b promotes cell proliferation, migration invasion, the EMT process and inhibits apoptosis.** KYSE-150 and KYSE-150R cells were transfected with miR-20b mimic or miR-20b inhibitor or their corresponding negative controls. (**A**) The cell proliferation assay was performed at the indicated time points. (**B**) Representative micrographs of cell migration assays (left) and the quantification (right). (**C**) Representative micrographs of cell invasion assays (left) and the quantification (right). (**D**) Representative micrographs of cell apoptosis assays (left) and the quantification (right). (**E**) Western blot analysis revealed that the E-cadherin expression level was decreased, while N-cadherin and Vimentin expression levels were elevated in cells transfected with miR-20b mimic. Data are shown as mean ± SD from three independent experiments. *P<0.05 by Student’s t-test.

**Figure 3:**
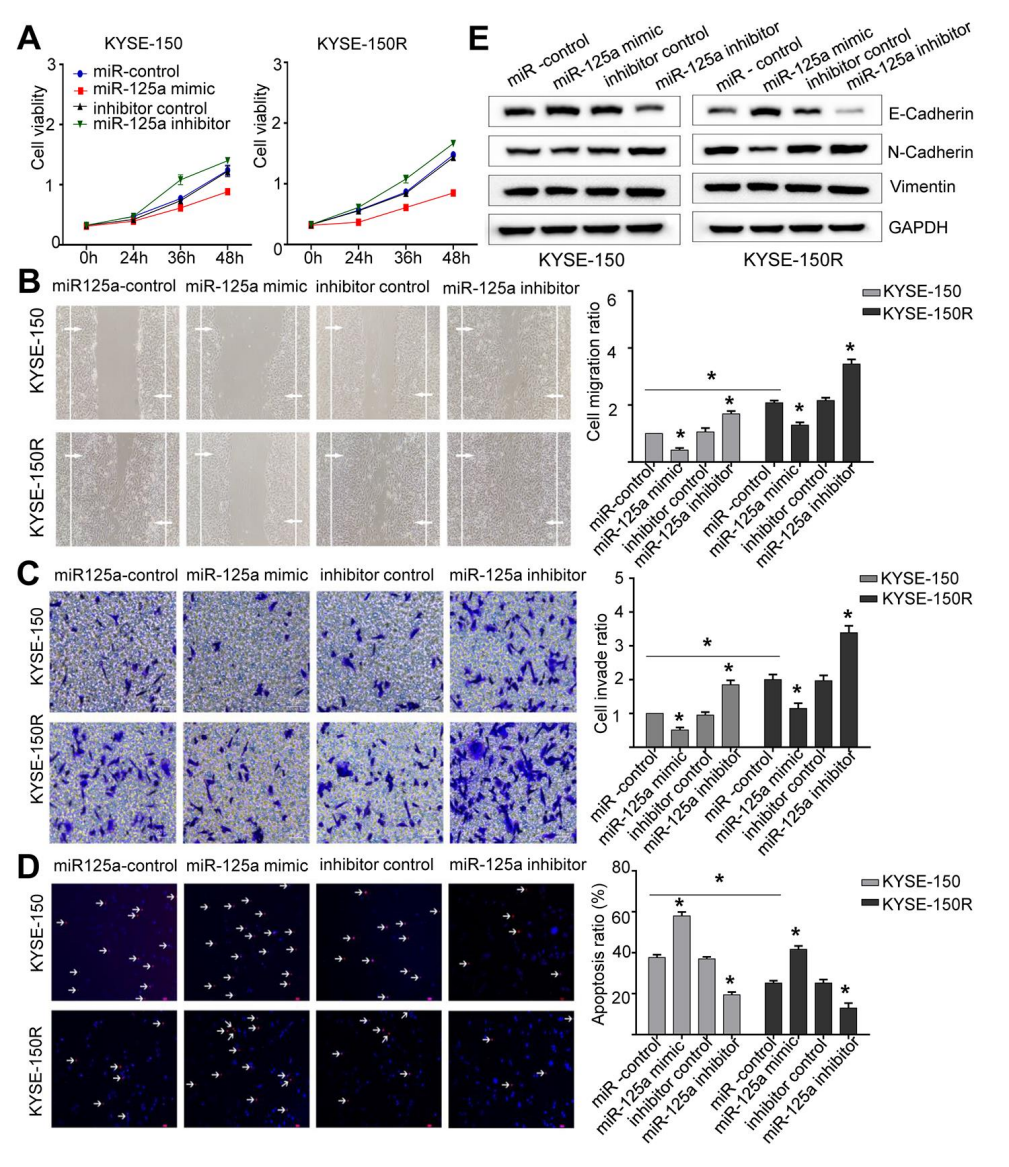
**miR-125a attenuates cell proliferation, migration invasion, the EMT process and induces apoptosis.** KYSE-150 and KYSE-150R cells were transfected with miR-125a mimic or miR-125a inhibitor or their corresponding negative controls. (**A**) The cell proliferation assay was performed at the indicated time points. (**B**) Representative micrographs of cell migration assays (left) and the quantification (right). (**C**) Representative micrographs of cell invasion assays (left) and the quantification (right). (**D**) Representative micrographs of cell apoptosis assays (left) and the quantification (right). (**E**) Western blot analysis revealed that the E-cadherin expression level was elevated, while N-cadherin and Vimentin levels were decreased in cells transfected with miR-125a mimic. Data are shown as mean ± SD from three independent experiments. *P<0.05 by Student’s t-test.

The results of comparison between KYSE-150 and KYSE-150R cells, indicated that latter exhibited a significant increase in cell proliferation, migration and invasion (Figures 2A–2C, 3A–3C). As miR-20b-5p was upregulated in KYSE-150R cells, we explored the miR-20b-5p function in cellular process. The results showed that overexpression of miR-20b-5p promoted cell proliferation, migration, and invasion. In contrast, downregulation of miR-20b-5p suppressed these cell activities in both KYSE-150 and KYSE-150R cells (Figure 2A–2C). Supporting Information Supplementary Figure 1A, 1B showed the transfection efficiency. TUNEL assay results revealed a significant decrease in the percentage of apoptotic cells in KYSE-150R cells compared to that in KYSE-150 cells (Figures 2D, 3D). In both cell lines, a reduction of apoptosis was found in miR-20b-5p overexpressing cells, whereas an enhancement of apoptosis was found in cells transfected with miR-20b-5p inhibitors (Figure 2D). Epithelial to mesenchymal transition (EMT) refers to a biological process during which epithelial cells lose cell junctions and adhesion and transit to an invasive mesenchymal phenotype [15]. Our previous research demonstrated that radioresistant EC cells were transformed to a

mesenchymal state [16]. In this study, we found that in both cell lines, overexpression of miR-20b-5p downregulated the E-cadherin protein, an epithelial marker, and upregulated N-cadherin and Vimentin proteins, which are mesenchymal markers (Figure 2E). These data indicated that miR-20b-5p promoted the EMT process in EC cells.

### MiR-125a-5p decreases cell proliferation, migration, invasion, and EMT process, and increases cell apoptosis

Since the expression of miR-125a-5p was lower in KYSE-150R cells (Figure 1B), we then explored the effect of miR-125a on the biological function of EC cells. We found that overexpression of miR-125a-5p inhibited cell proliferation, migration and invasion, whereas miR-125a-5p inhibitor exhibited a reverse effect in both KYSE-150 and KYSE-150R cells (Figure 3A–3C). The transfection efficiency was shown in Supporting Information Supplementary Figure 1C, 1D. The results of TUNEL assays demonstrated that cell apoptosis was accelerated by transfection with miR-125a-5p mimics and suppressed by transfection with miR-125a-5p inhibitors (Figure 3D). As for EMT process, in both cell lines, overexpression of miR-125a-5p increased E-cadherin protein expression levels, and reduced N-cadherin and Vimentin protein expression levels compared to those in the control group (Figure 3E). These results suggested that miR-125a-5p inhibited EMT in EC cells.

### PTEN is a downstream target of miR-20b-5p, while IL6R is a downstream target of miR-125a-5p

**Figure 4:**
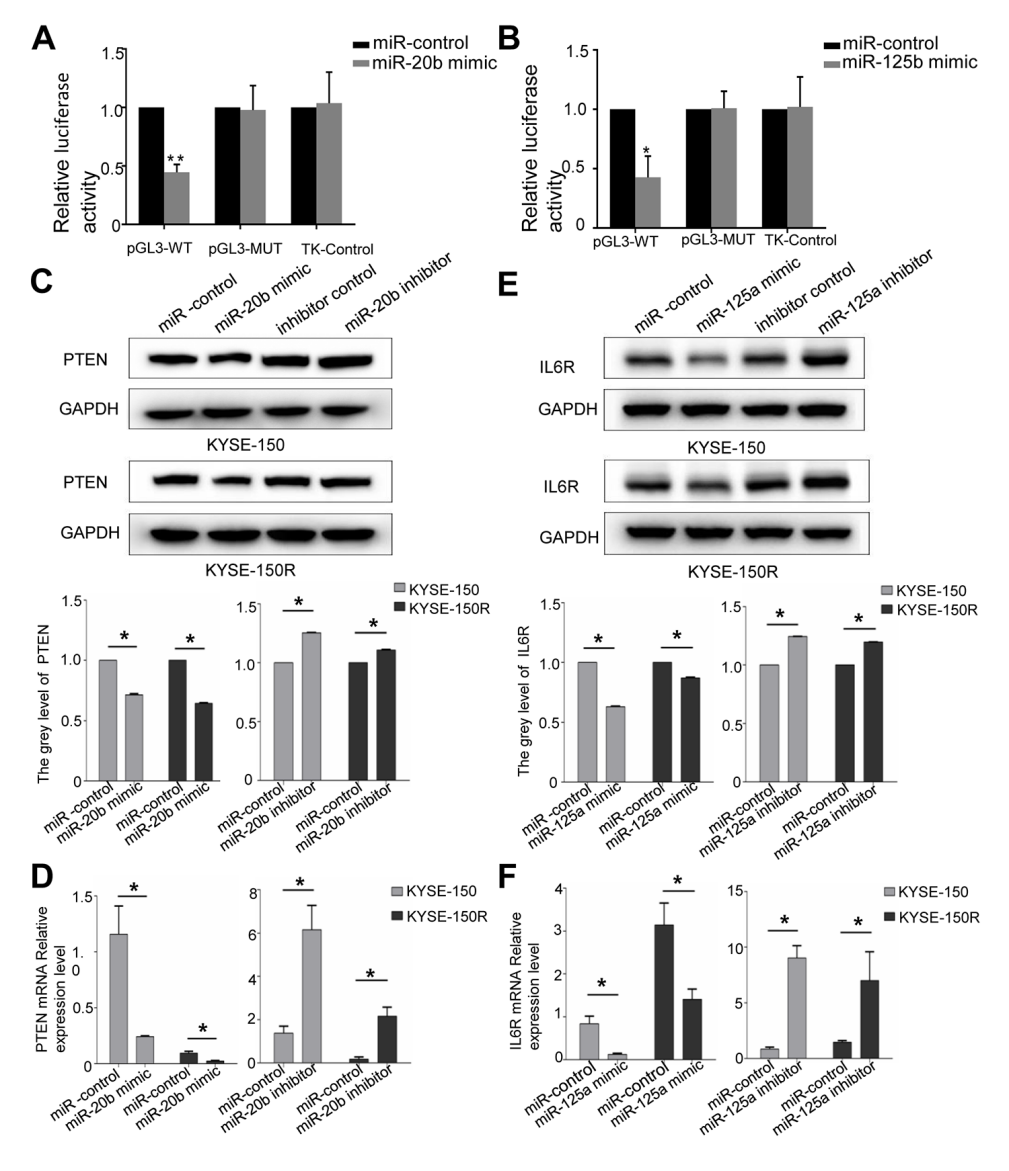
**PTEN is a target of miR-20b, IL6R is a target of miR125a.** (**A**) Analysis of the luciferase reporter assay. Firefly luciferase reporters containing either WT or MUT miR-20b binding sites in the PTEN 3’-UTR were co-transfected into KYSE-150 and KYSE-150R cells with miR-20b and miR-20b control. (**B**) Analysis of the luciferase reporter assay. Firefly luciferase reporters containing either WT or MUT miR-125a binding sites in the IL6R 3’-UTR were co-transfected into cells with miR-125a and miR-125a control. (**C**, **D**) Western blot (**C**) and RT-PCR (**D**) analysis of PTEN expression level in cells transfected with miR-20b mimic or inhibitor. (**E**, **F**) Western blot (**E**) and RT-PCR (**F**) analysis of IL6R expression level in cells transfected with miR-125a mimic or inhibitor. *P<0.05 by Student’s t-test. MUT, mutant; WT, wild type; GAPDH, glyceraldehyde 3-phosphate dehydrogenase.

Based on the miRNA database TargetScan and miRanda, the predicted targets of miR-20b and miR-125a are PTEN and IL6R, respectively (Supporting Information Supplementary Figure 2A, 2B). Therefore, we constructed PTEN and IL6R reporter vectors to perform dual-luciferase reporter assays. In KYSE-150R cells, miR-20b-5p mimics significantly inhibited PTEN luciferase activity (Figure 4A). However, a mutation in the binding site of miR-20b-5p in PTEN 3'-UTR abrogated the effects of miR-20b-5p on the luciferase activity. The co-transfection of miR-125a-5p mimic and IL6R WT 3'-UTR remarkably reduced the luciferase activity in KYSE-150R cells. The phenomenon was not observed after miR-125a-5p mimic and IL6R Mut 3'-UTR co-transfection (Figure 4B). Western blot as well as RT-PCR analysis proved that overexpression of miR-20b-5p and miR-125a-5p could negatively regulate the expression of PTEN and IL6R at both mRNA and protein levels (Figure 4C–4F), respectively. Previous studies discovered that PTEN/Akt signaling pathway and IL-6R/JAK/STAT pathway are associated with carcinoma tumorigenesis [17, 18]. Our results showed that miR-20b-5p mimics did not change the protein level of Akt and ERK in KYSE-150 cells, but remarkably increased p-Akt and p-ERK expression compared with miR-20b mimics-NC (Supporting Information Supplementary Figure 3). MiR-125a-5p mimics significantly down-regulated p-STAT3 and p-JAK2 expression compared with miR-125a-5p mimics-NC (Supporting Information Supplementary Figure 4). These data verified that miR-20b-5p and miR-125a-5p directly target PTEN and IL6R in EC, respectively.

### Restored PTEN expression could reverse the changes caused by miR-20b-5p overexpression in EC cells

**Figure 5:**
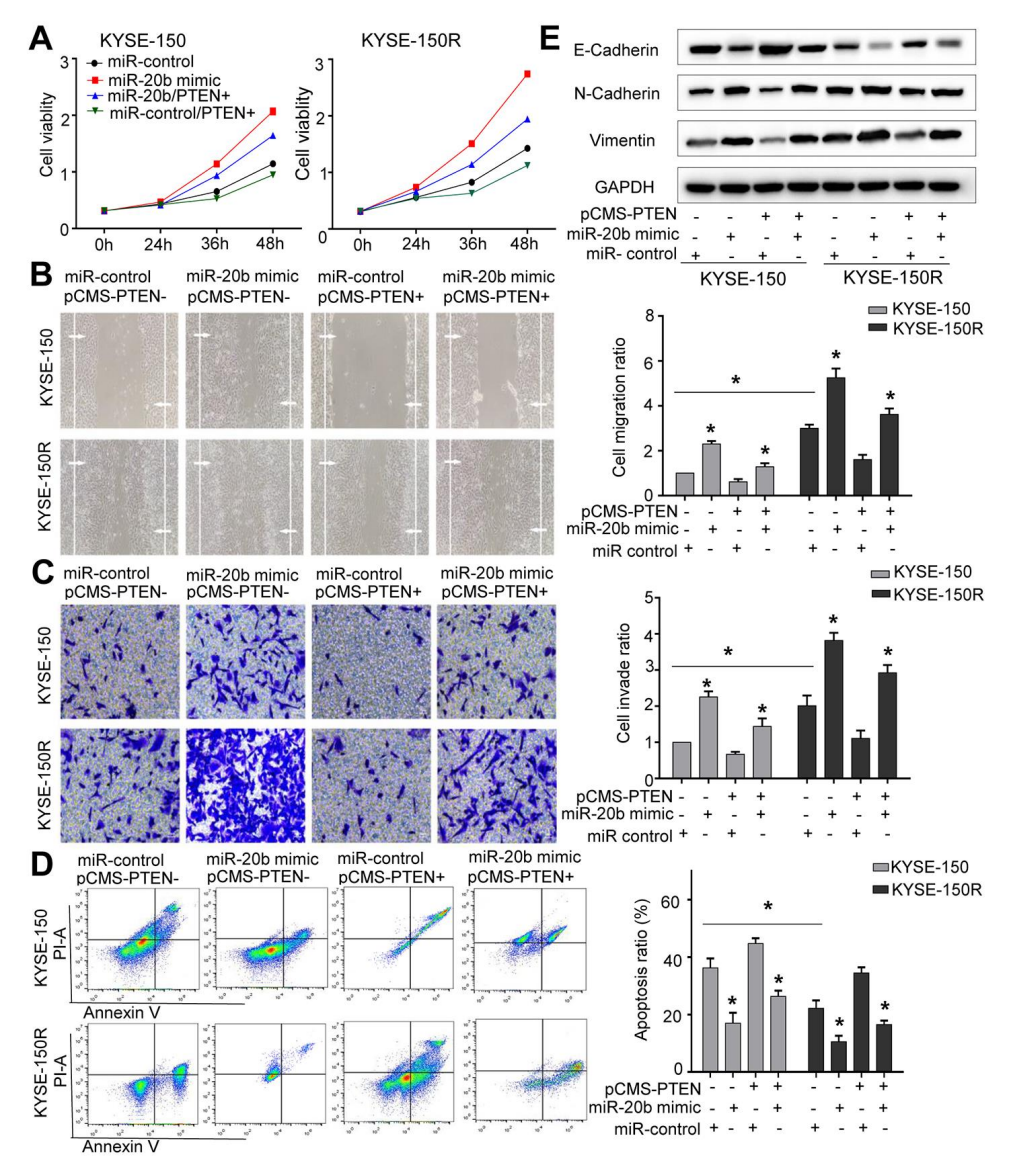
**The effects of miR-20b and PTEN on KYSE-150 and KYSE-150R cells.** KYSE-150 and KYSE-150R cells were transfected with miR-con or miR-20b mimic, or co-transfected with PTEN and miR-con/miR-20b mimic. (**A**) The cell proliferation assay was performed at the indicated time points. (**B**) Representative micrographs of cell migration assays (left) and the quantification (right). (**C**) Representative micrographs of cell invasion assays (left) and the quantification (right). (**D**) Representative micrographs of cell apoptosis assays (left) and the quantification (right). (**E**) Western blot analysis revealed that transfection of PTEN inhibited the EMT process and co-transfection of miR-20b mimic and PTEN promoted the EMT process. Data are shown as mean ± SD from three independent experiments. *P<0.05 by Student’s t-test.

To clarify whether miR-20b-5p increased cellular growth and migration controlled by PTEN, both cell lines were co-transfected with miR-20b-5p mimics and PTEN. Efficient overexpression of PTEN was achieved as shown in Supporting Information Supplementary Figure 5A. As expected, co-transfection with PTEN reduced cell proliferation (Figure 5A), migration (Figure 5B) and invasion abilities (Figure 5C) (all of which were increased by miR-20b-5p mimics), and increased cell apoptosis (Figure 5D). Western blot analysis showed that in KYSE-150R cells, transfection of PTEN decreased the EMT process (Figure 5E). Based on these data, we proved that miR-20b-5p increased KYSE-150R cell growth and metastasis by targeting PTEN.

### Restored IL6R expression could reverse the changes caused by miR-125a-5p overexpression in EC cells

**Figure 6:**
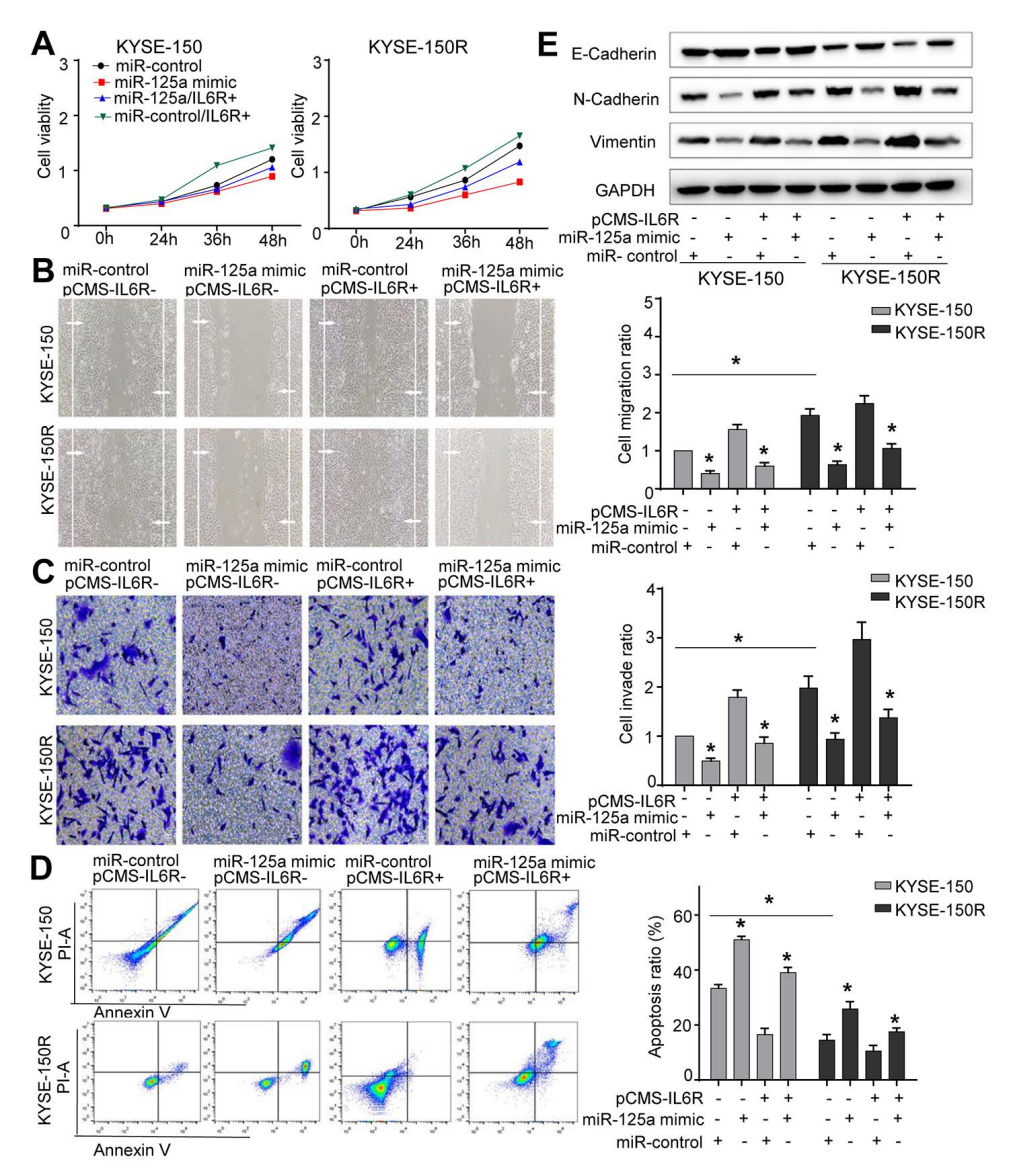
**The effects of miR-125a and IL6R on KYSE-150 and KYSE-150R cells.** KYSE-150 and KYSE-150R cells were transfected with miR-con or miR-125a mimic, or co-transfected with IL6R and miR-con/miR-125a mimic. (**A**) The cell proliferation assay was performed at the indicated time points. (**B**) Representative micrographs of cell migration assays (left) and the quantification (right). (**C**) Representative micrographs of cell invasion assays (left) and the quantification (right). (**D**) Representative micrographs of cell apoptosis assays (left) and the quantification (right). Data are shown as mean ± SD from three independent experiments. (**E**) Western blot analysis revealed that transfection of IL6R promoted the EMT process and co-transfection of miR-125a mimic and IL6R attenuated the EMT process. *P<0.05 by Student’s t-test.

Efficient overexpression of IL6R was achieved as shown in Supporting Information Supplementary Figure 5B. Co-transfection with miR-125a-5p mimics and IL6R in both cell lines improved cell proliferation (Figure 6A), migration (Figure 6B) and invasion abilities (Figure 6C), and decreased cell apoptosis (Figure 6D) that was altered by miR-125a-5p mimic. Western blot analysis showed that transfection of KYSE-150R cells with IL6R, promoted the EMT process (Figure 6E). Thus, our results suggested that miR-125a-5p decreased KYSE-150R cell growth and metastasis by targeting the IL6R.

## DISCUSSION

Radiation therapy has been suggested as an efficient treatment in patients with EC. Enhancement of tumor response to radiotherapy and reduction of radioresistance are crucial for improving the treatment outcome of EC. MiRNAs have been recognized for their role in mediation of the radiation resistance in tumors [19].

In current study, we firstly verified that the expression level of miR-20b-5p was markedly elevated in KYSE-150R cells compared to that in KYSE-150 cells. MiR-20b has been verified to increase cell aggressiveness via regulation of PTEN expression in EC and prostate cancer [20, 21]. Consistent with these studies, we observed increased cell proliferation, invasion, and migration in cells overexpressing miR-20b-5p. Apoptosis is one of the mechanisms of tumor death induced by radiotherapy. Apoptosis resistance is one of the characteristics of tumors and leads to cancer progression [22]. EMT has also been verified to be relevant to tumor invasion and metastasis [13]. Recent reports have indicated that EMT was also associated with therapy resistance [23]. Several miRNAs have been demonstrated to affect the EMT process in cancers [24]. MiR-21 was reported to improve transformation of EMT in drug-resistant lung adenocarcinoma cancer cells [25]. Our findings suggested that miR-20b-5p inhibited cell apoptosis and promoted EMT process in EC cells.

Moreover, we found miR-20b-5p exerted its function on KYSE-150R cells by targeting PTEN, which is among the most frequently transformed tumor-suppressor genes in human cancers. PTEN has been reported to inhibit oncogenic PI3K activation, and subsequently block the PI3K/Akt signaling pathway, which is known to induce tumor cell progression [26]. Wu et al. reported that miR-21 promoted EC cell proliferation, migration through the PTEN/PI3K/AKT signaling pathway. Loss of PTEN leads to a continuous activation of the signal pathway, and therefore, fail to control cell growth [17]. Kohnoh et al. demonstrated that unphosphorylated PTEN could inhibit hypoxia-induced EMT in lung cancer cells [27]. Low expression of PTEN has also been reported correlates with poor clinical outcomes of various human cancers [28], as well as plays a role in resistance of anti-tumor treatment. Chen et al. demonstrated that miR-21-5p conferred doxorubicin resistance in gastric cancer cells by targeting PTEN [29]. Wu et al. verified that miR-222 conferred radioresistance in nasopharyngeal carcinoma through modulating PTEN expression [30]. Thus, all these results supported the important role of PTEN in cancer cell development. Results of current study indicate miR-20b-5p exerts its function partly through PTEN /PI3K/Akt signaling pathway.

Our study also found that there was a low expression level of miR-125a-5p in radioresistant KYSE-150R cells. Zhang et al. reported that miR-125a suppressed bladder cancer cell proliferation by inducing cell cycle arrest and cell apoptosis. Cell cycle became stagnant at the G0/G1 phase when miR-125a-5p was overexpressed [31]. In cervical cancer, cell invasion and tumor metastasis has been reported to be regulated by miR-125a-5p as it targets STAT3 [32]. Current study results showed that miR-125a-5p weakened cell proliferation, migration, invasion, and the EMT process. In addition, miR-125a-5p also increased apoptosis via IL6R, an oncogene which encodes a subunit of the interleukin 6 (IL6) receptor complex [33]. Combining IL6 and IL6R leads to the activation of several signal transduction pathways including the JAK/STAT, PI3K/Akt and MAPK pathways [34]. High levels of IL6 in several cancer cells, such as prostate cancer, ovarian cancer, and renal cell carcinoma, resulted in poor prognosis in cancer patients [35–37]. Overexpression of IL6R has been demonstrated to correlate with the increase in proliferative activity and the inhibition of apoptosis [38]. Rokavec et al. reported that the activation of IL-6R/STAT3/miR-34a loop was not only necessary for invasion and metastasis of colorectal cancer cells, but also associated with lymph node metastasis and distant metastasis in patient samples. Silencing IL6R expression suppressed tumor growth, migration and angiogenesis, as well as enhanced the antitumor activity in several cancer cells [39]. In pancreatic ductal adenocarcinoma, application of IL6R blocking antibodies through inhibiting IL6 signaling could shift the tumor microenvironment from a chemoresistant state to a chemosensitive state, which was accompanied with reduced STAT3 activation [40]. Results of current study indicate that miR-125a-5p exerts its function via IL6R to activate JAK-STAT3 signaling pathway. Combined with the previous study, our data suggested that miR-125a-5p served as an important tumor suppressor gene in EC cells.

## MATERIALS AND METHODS

### Cell culture and reagents

Human esophageal squamous cancer cell line KYSE-150 was purchased from the Cell Bank of Type Culture Collection of Chinese Academy of Sciences (Shanghai, China). Our department has already established the radioresistant cell line KYSE-150R using a gradient dose irradiation treatment [17]. Cells were cultured in RPMI-1640 medium (Gibco, Gaithersburg, MD, USA) supplemented with 10% fetal bovine serum (FBS) (Gibco) and 1% penicillin/streptomycin solution (Gibco) in an atmosphere of 5% CO2 at 37° C. The cell lines were subcultured every 2–3 days following digestion at room temperature with 0.5 mL trypsin/EDTA per well (Sigma-Aldrich Ltd, UK).

The primers were synthesized by the Genewiz (Suzhou, China). The miRNAs and plasmids were obtained from GenePharma (Shanghai, China). The primer sequences were listed as following:

PTEN: 5’-CACAGAATTCCAGACATGACAGCCATCATC-3’ and 5’-GTGGATCCTCATGGTGTTTTATCCCTCTTG-3’;

IL6R: 5’-CGCGAATTCATGATTGACAAACAAATTC-3’and 5’-CCGGATCCTTACATTTGCCGAAGA-3’;

β-actin: 5’-ACACTGTGCCCATCTACGAGG-3’ and 5’-AGGGGCCGGACTCGTCATACT-3’.

### Quantitative real-time PCR (qRT-PCR)

TRIzol reagent (Invitrogen, Gaithersburg, MD, USA) was utilized to obtain RNA from cultured cells. qPCR was carried out in an ABI 7500 instrument (Thermo Scientific, Waltham, MA, USA) using the TaqMan™ MicroRNA Assay (Thermo Scientific, Waltham, MA, USA) for miRNA and Takara® CellAmp™ Direct SYBR® RT-qPCR Kit (Takara, Kusatsu, Japan) for cDNA synthesis.

### MiRNA and cell transfection

GenePharma provided (Shanghai, China) miR-20b-5p mimic, miR-125a-5p mimic, miR-20b-5p inhibitor, miR-125a-5p inhibitor and their corresponding negative controls. Lipofectamine 2000® (Invitrogen, Carlsbad, CA, USA) was used for cell transfection following the manufacturer’s instructions.

### Cell viability analysis

Cell proliferation was determined using Cell Counting Kit-8 (Beyotime Biotechnology, Shanghai, China). Cells were seeded into 96-well plates at a density of 3×10^3^ cells per well. At 24, 36 and 48 hours post transfection, 20 μL of CCK-8 solution was added to each well. After a 4-hour incubation, the absorbance values were determined at 450 nm.

### Cell migration assay

Wound-healing assay was used to assess cell migration ability. EC cells, after transfection treatment, were seeded in 24-well plates and then cultured for 24 h until 90% confluence was obtained. In each well, a scratch in a confluent monolayer was created using a 100 μL pipette tip and cultured for 24 h. The cells were photographed at 0 and 24 h under a Nikon Eclipse TE2000-U Inverted Microscope (Nikon, Tokyo, Japan).

### Cell invasion assay

Cell invasion ability was examined using a transwell chamber (Corning, NY, USA). After coating the upper chamber with Matrigel, 3 × 10^4^ transfected cells in serum-free DMEM medium were transferred to the upper chamber. In the lower chamber, 500 μL of the complete medium with 10% FBS was added. After 24 h of incubation, a cotton swab was used to wipe non-invading cells. The invaded cells were fixed with 4% paraformaldehyde, stained with 1% crystal violet solution (Sigma, St. Louis, MO, USA) and counted in five randomly selected fields under a light microscope.

### Cell apoptosis

### *Terminal deoxynucleotidyl transferase dUTP nick end labeling (TUNEL) assay*

TUNEL assay was performed using the Cell Death Detection Kit (Roche, Mannheim, Germany) according to the manufacturer's instructions. Briefly, 1 × 10^5^ cells were seeded in a six-well plate 48 h post-transfection, then washed with PBS and fixed with the buffer provided in the kit. DAPI was used to label cell nuclei for 10 minutes. Under the fluorescence microscope, cells with a dark green fluorescence were defined as apoptotic cells.

### *Annexin V–FITC assay*

Annexin V–FITC assay was used to detect apoptosis. The transfected cells were seeded in six-well plates at a density of 1 × 10^5^ per well 48 h post-transfection. After washing with PBS, 10 μL of Annexin V–FITC (ab14085, Abcam, Cambridge, MA, USA) and 5 μL of propidium iodide were added to each well and mixed gently. After 15-minutes, 300 μL of the binding buffer was added and cell apoptosis was detected using FACSCalibur^TM^ (BD Biosciences, San Jose, CA, USA).

### Western blot

Total proteins were extracted using RIPA buffer (Thermo Scientific, Waltham, MA, USA) following the manufacturer’s protocol, separated by SDS-polyacrylamide gel electrophoresis and transferred to polyvinylidene fluoride membrane (MilliporeSigma, Burlington, MA, USA). The blots were incubated with primary antibodies, followed by the incubation with the appropriate horse radish peroxidase conjugated secondary antibody, detected with Bio-Rad® ChemiDoc® MP(Bio-Rad), and were analyzed using Quantity One 4.6 software (Bio-Rad Laboratories, Inc., Hercules, CA, USA). The primary antibodies used included anti-E-cadherin (cat. no. 1416, Abcam, Cambridge, United Kingdom), anti-N-cadherin (ab18203, Abcam, Cambridge, MA, USA), anti-Vimentin (ab8978, Abcam, Cambridge, MA, USA), anti-GAPDH antibody (ab8245, Abcam, Cambridge, MA, USA), anti-PTEN antibody (ab32199, Abcam Cambridge, MA, USA), anti-IL6R antibody (ab128008, Abcam, Cambridge, MA, USA), anti-Akt antibody (10176-2-AP, proteintech, Wuhan, China), anti-p-Akt antibody (66444-1-Ig, proteintech), anti-ERK antibody (16443-1-AP, proteintech), anti-p-ERK antibody (#3510, Cell Signaling Technology, Danvers, MA, USA) anti-p-STAT3 antibody (#9145, Cell Signaling Technology), anti-p-JAK2 antibody (#3771, Cell Signaling Technology), and anti-β-actin antibody (ab8226, Abcam).

### Dual-luciferase reporter gene assay

A wild-type 3’-UTR and a mutant 3’-UTR of PTEN and IL6R were amplified from KYSE-150 cells using PCR, and cloned into a pGL3-Basic vector. Cells were co-transfected with the miRNAs, firefly luciferase reporter vector and renilla luciferase control vector using Lipofectamine 2000 according to the manufacturer’s instructions. All cells were incubated for 48 hours and assayed using a luciferase reporter assay system (Promega, Madison, WI, USA). Renilla luciferase activity was regarded as an internal control.

### Statistical analysis

SPSS version 20.0 statistical software (SPSS, Chicago, IL, USA) was used for statistical analyses. Experimental data were calculated as mean ± standard deviation of at least three independent assays. The Student *t*-test was conducted to compare the difference between the two groups. P < 0.05 denoted statistical significance.

## Supplementary Material

Supplementary Figures
