# Supplementary material for: miR-20b and miR-125a promote tumorigenesis in radioresistant esophageal carcinoma cells
Source: Aging (Albany NY). 2021 Mar 10;13(7):9566–81. doi: 10.18632/aging.202690 (PMC8064182; doi:10.18632/aging.202690)
Supplement: Supplementary Figures [file aging-13-202690-s001.docx]

Supplementary figures


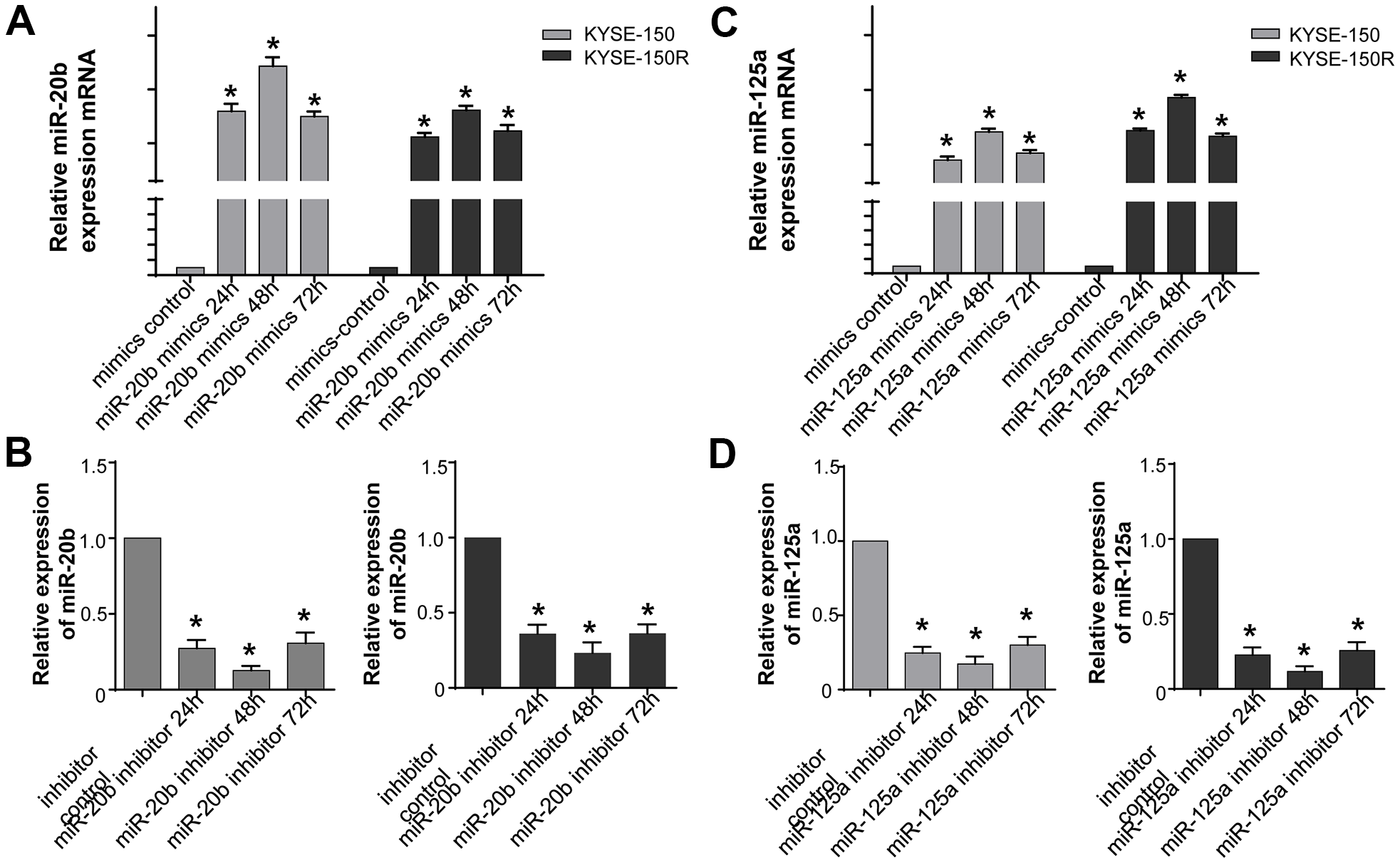


Supplementary Figure 1. Analysis of transfection efficiency in KYSE-150 and KYSE-150R cells of mimics and inhibitors of miR-20b and miR-125a. (A) The relative expression of miR-20b in KYSE-150 and KYSE-150R cells treated with miR-20b mimics and mimics controls were analyzed by qPCR following transfection for 24h, 48h, 72h. (B) The relative expression of miR-125a in KYSE-150 and KYSE-150R cells treated with miR-125a mimics and mimics controls following transfection for 24h, 48h, 72h. (C) The relative expression of miR-20b in KYSE-150 (left) and KYSE-150R cells (right) treated with miR-20b inhibitors and inhibitors controls following transfection for 24h, 48h, 72h. (D) The relative expression of miR-125a in KYSE-150 (left) and KYSE-150R cells (right) treated with miR-125a inhibitors and inhibitors controls following transfection for 24h, 48h, 72h. *P<0.05.


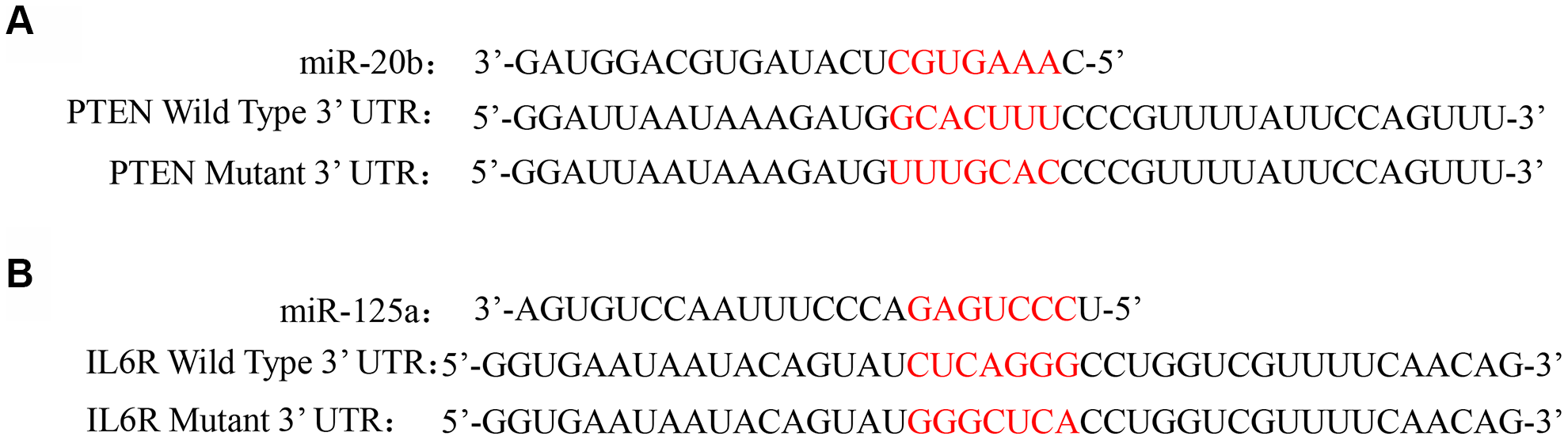


Supplementary Figure 2. PTEN is a target of miR-20b, IL6R is a target of miR125a. (A) Position of the miR-20b target site in 3′-UTR of PTEN predicted by TargetScan and miRanda. (B) Position of the miR-125a target site in 3′-UTR of IL6R predicted by TargetScan and miRanda.


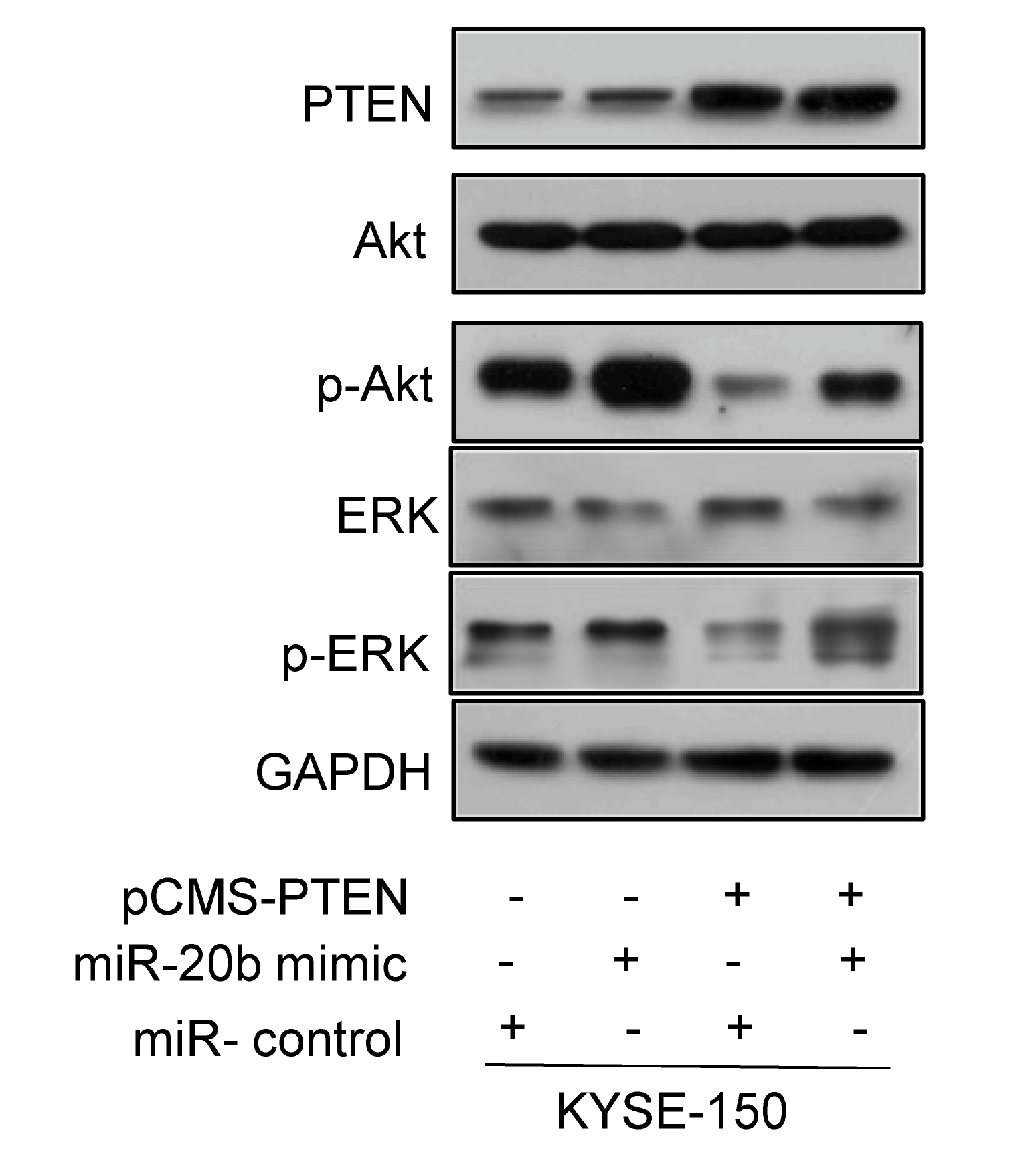


Supplementary Figure 3. miR-20b-5p regulates PI3K/Akt and MAPK pathways by targeting PTEN. The protein expression of PTEN, Akt, p-Akt, ERK and p-ERK in KYSE-150 cells co-transfected with miR-con/miR-20b and pcDNA3.1/pCMS-PTEN was measured by western blot.


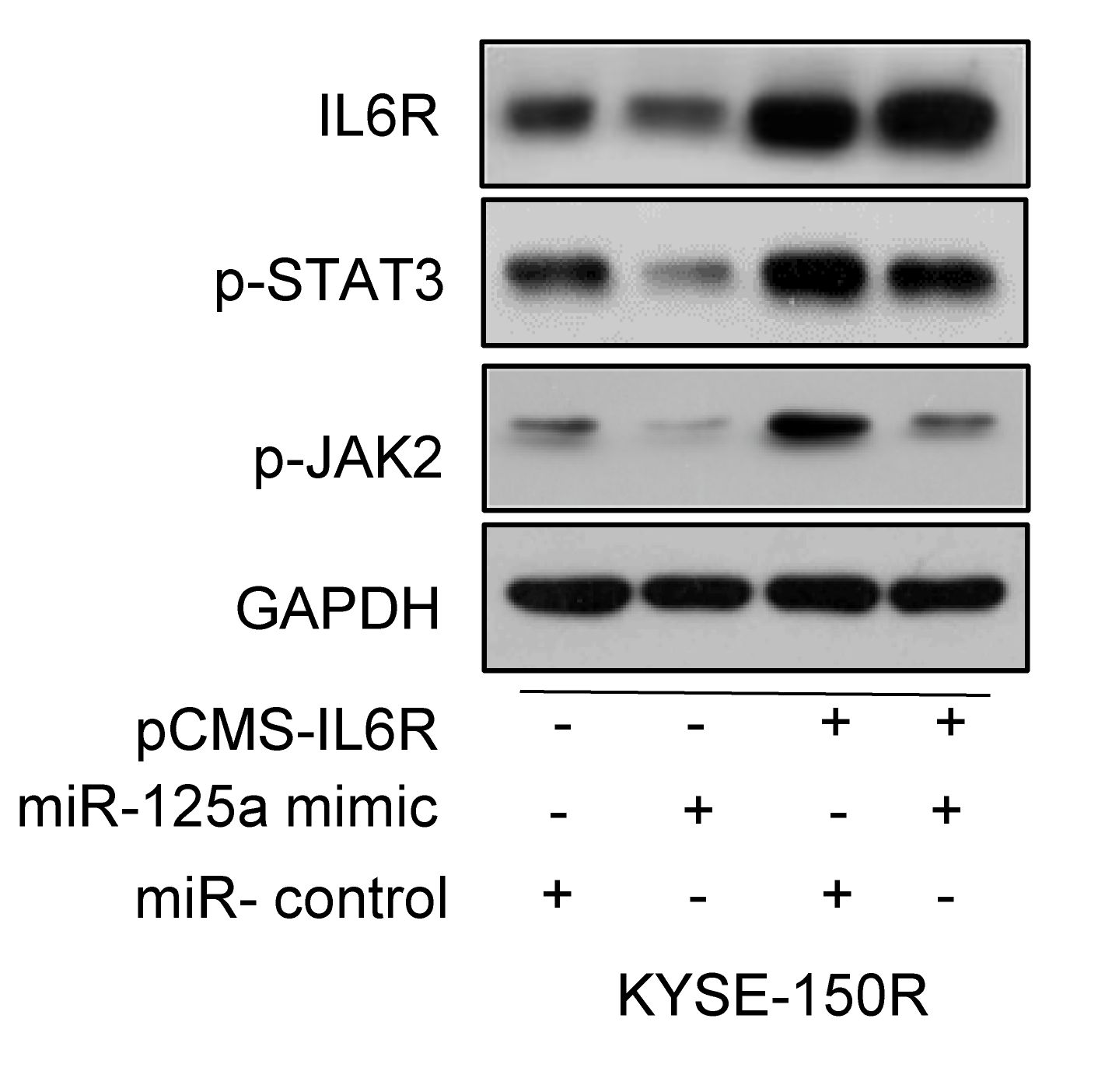


Supplementary Figure 4. miR-125a-5p regulates the JAK-STAT3 signaling pathway by targeting IL6R. The protein expression of IL6R, p-STAT3 and p-JAK2 in KYSE-150R cells co-transfected with miR-con/miR-125a and pcDNA3.1/pCMS-IL6R was measured by western blot.


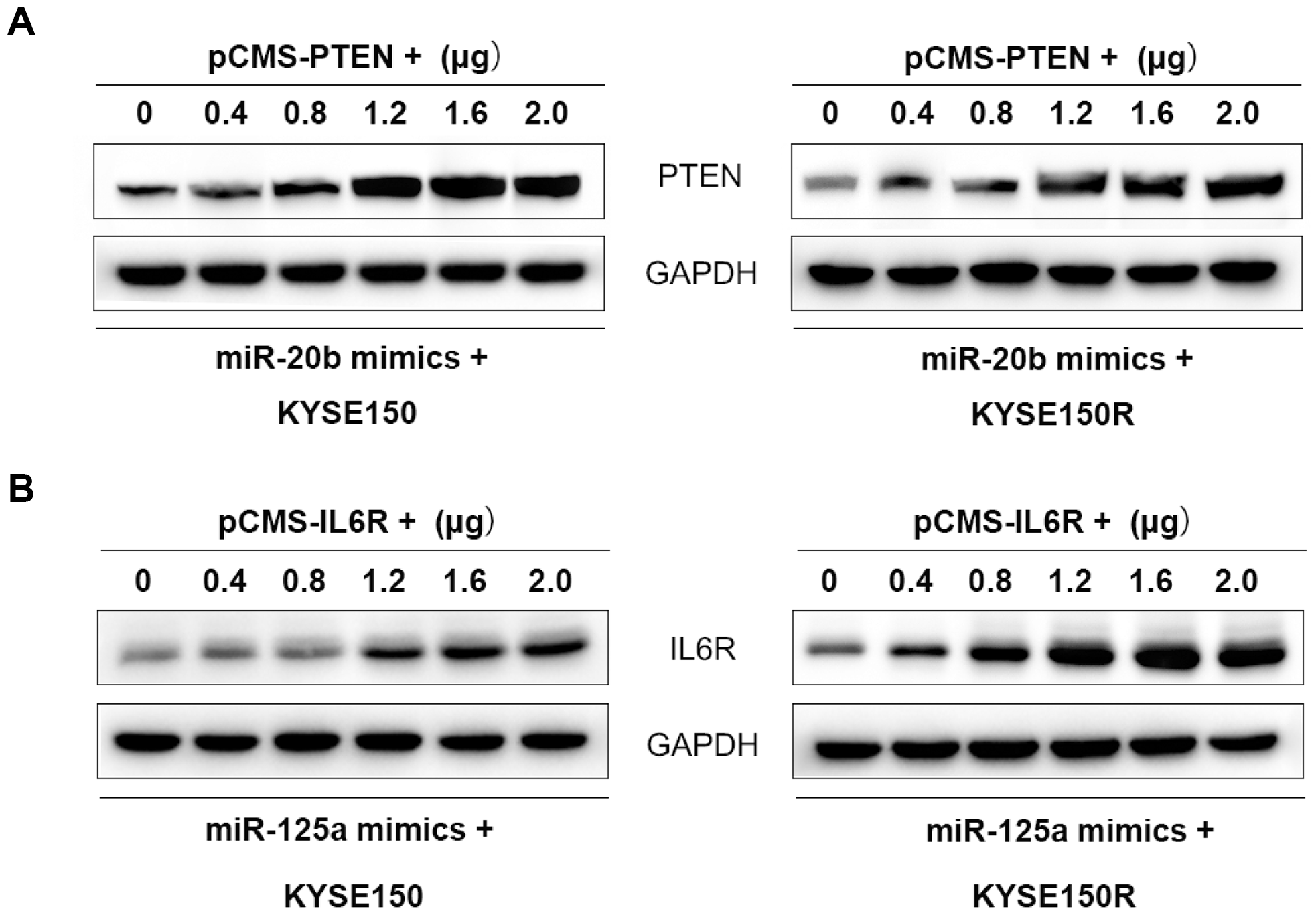


Supplementary Figure 5. Analysis of transfection efficiency in KYSE-150 and KYSE-150R cells of PTEN vector and IL6R vector. (A) The protein expression of PTEN transfected with PTEN vector measured by western blot. (B) The protein expression of IL6R transfected with IL6R vector measured by western blot.
